# Mapping self-awareness of cancer-related cognitive impairment: a scoping review of evidence, methods, and neurobiological correlates

**DOI:** 10.3389/fneur.2025.1662935

**Published:** 2025-12-11

**Authors:** Davide Spinetti, Mariaines Orzelleca

**Affiliations:** Department of General Psychology (DPG), University of Padua, Padua, Italy

**Keywords:** scoping review, anosognosia, CRCI, metacognition, cancer survivor, neuropsychology, neuroimaging

## Abstract

**Background:**

Cancer-related cognitive impairment (CRCI) is a frequent and distressing side effect among cancer survivors. While many patients report persistent cognitive difficulties, a notable discrepancy often exists between subjective complaints and objective performance on neuropsychological testing. This gap raises critical questions about self-awareness and metacognitive insight in the context of CRCI. Despite the clinical relevance of this phenomenon, conceptualized as anosognosia in other neurological conditions, its presence in oncology remains insufficiently explored.

**Objective:**

This scoping review aims to map the existing literature on self-awareness of cognitive impairment in cancer survivors, with a focus on studies examining the discrepancy between subjective and objective cognition, the methodologies used to assess awareness, and the clinical and theoretical implications of impaired metacognition in this population.

**Methods:**

A systematic search was conducted on PubMed for articles published between 2000 and 2025. Inclusion criteria comprised peer-reviewed studies involving adult cancer survivors that investigated subjective and/or objective cognitive functioning, and addressed aspects of self-awareness, metacognitive monitoring, or anosognosia. Studies were screened, selected, and charted following PRISMA-ScR guidelines.

**Results:**

Forty six studies met the inclusion criteria. Most reported a weak or inconsistent correlation between self-reported and objectively measured cognition. A minority employed formal tools to assess metacognitive accuracy or insight. Methodological heterogeneity and a lack of consensus in terminology (e.g., “awareness,” “insight,” “complaints”) limited cross-study comparisons. Only a small number of articles conceptualized this discrepancy in relation to anosognosia or broader models of self-awareness. Factors such as age, mood symptoms, fatigue, and neurobiological correlates (e.g., alterations in the default mode network) were identified as potential moderators of impaired awareness.

**Conclusion:**

Despite growing evidence of subjective-objective cognitive discrepancies in cancer survivors, the construct of self-awareness remains under-theorized and inconsistently measured in the literature. There is an urgent need for standardization of terms and tools, and for theoretically informed approaches to capture metacognitive impairment in this context. Greater clarity in this domain may inform more tailored interventions, improve survivorship care, and advance the neuropsychological understanding of CRCI.

## Introduction

Cancer-related cognitive impairment (CRCI), commonly referred to as “chemobrain” or “chemofog,” affects a substantial portion of cancer survivors and has become a prominent area of concern in survivorship care (1–3). Self-reported prevalence rates vary widely, from approximately 16 to 60%, depending on treatment modality, cancer type, and time elapsed since treatment completion (4, 5). Importantly, large epidemiological studies have shown that cancer survivors are significantly more likely to report cognitive complaints, including memory issues, compared to non-cancer controls (6–10). Although the literature often links adjuvant chemotherapy to cognitive deterioration, a study by Kim et al. (11) shows that in patients over 65, chemotherapy is not a significant predictor of cognitive complaints 5–6 years after diagnosis. This suggests a greater role for factors such as age, mood, and premorbid cognitive reserves. Contrary to early assumptions, CRCI is not a phenomenon restricted to elderly patients. Emerging research has demonstrated that younger survivors, especially those under 55 years, often report greater cognitive distress and perceive the impact of CRCI more acutely, particularly in work-related and social domains (12–14). The symptoms of CRCI are also multifaceted, extending beyond memory to include impairments in attention, processing and motor speed, language, working and long term memory and executive functioning (2, 15–22). These symptoms have been reported following a wide array of treatments, including endocrine therapy, chemotherapy, hormonal therapy, radiotherapy, targeted therapy, thyroidectomy and more recently, immunotherapy (16, 22–26). Despite the growing attention to CRCI, a key challenge remains the apparent disconnection between subjective and objective indicators of cognitive functioning. Many survivors report considerable difficulties in daily life, such as struggling to focus, multitask, or remember appointments, yet perform within normal ranges on standardized neuropsychological assessments (27). Conversely, some individuals demonstrate measurable cognitive deficits but report no significant complaints, raising questions about the accuracy of self-perception. This discrepancy highlights the need to explore concepts significantly discussed in neuropsychology, particularly *anosognosia*, or impaired awareness of cognitive impairment. Anosognosia, initially described by (96) in hemiplegic stroke patients, is now recognized across various neurological conditions, including traumatic brain injury (TBI), Alzheimer’s disease, and frontotemporal dementia (28–30). It is typically attributed to disruptions in metacognitive systems responsible for self-monitoring, error detection, and self-reflection (31, 32). In the context of CRCI, a lack of awareness may have significant consequences: survivors who underestimate their difficulties may be less likely to seek support, adhere to interventions, or communicate cognitive needs in clinical settings. Despite its relevance, the concept of anosognosia remains largely underexplored in the oncology field. Few studies have attempted to operationalize or measure self-awareness in cancer survivors, and those that do often use inconsistent terminology or indirect assessments. Qualitative studies and focus groups have begun to illuminate survivors’ lived experiences, coping strategies, and beliefs about cognitive change. However, these insights remain fragmented and largely descriptive (13, 33). Given these gaps, there is a clear need for a structured synthesis of the existing literature. The aim of this scoping review is to map the current evidence on self-awareness of cognitive impairment in cancer survivors. Specifically, we sought to identify and describe studies that: (a) compare subjective complaints with objective cognitive measures; (b) assess awareness explicitly using standardized tools or definitions; (c) discuss theoretical frameworks and clinical consequences related to awareness or unawareness; and (d) highlight key gaps in the literature and future directions for research. This review, therefore, aims to clarify how the concept of anosognosia, well-studied in neurology and psychiatry, may be relevant to oncology and survivorship care, and to identify whether survivors’ lack of insight is being sufficiently acknowledged, measured, and addressed in existing research. By doing so, we aim to provide the groundwork for future empirical research.

## Methods: framework and guidelines

This scoping review was conducted following the Preferred Reporting Items For Systematic Reviews and Meta-Analyses extension for Scoping Reviews (PRISMA-ScR) checklist (34). Although the protocol was not registered prospectively on platforms such as Open Science Framework (OSF) or PROSPERO, the methodological steps were defined *a priori* and adhered to throughout the review process.

### Database and search strategy

A comprehensive literature search was conducted in PubMed. The search included articles published in English between January 2000 and July 2025, to reflect the modern era of cancer survivorship and cognitive neuroscience, as well as broader neurological contexts relevant to cognitive awareness. While broad terms like “cancer survivors” or “anosognosia” or “cancer related cognitive impairment” alone can yield a very large number of results, a specific Boolean search string was designed to be highly focused, combining terms related to four key conceptual areas with ‘AND’ operators to ensure high specificity. The full Boolean string used for the search was: ((“cancer survivors” OR “oncology patients”) AND (“cognitive impairment” OR “chemobrain” OR CRCI OR “cognitive dysfunction” OR “cognitive decline”)) AND (anosognosia OR “awareness of deficits” OR metacognition OR insight) AND (“self report” OR “neuropsychological testing” OR “subjective cognition” OR “objective cognition” OR discrepancy OR mismatch). This rigorous combination of keywords, along with the specified date range and language filter, resulted in a manageable initial pool of records for screening. Although the initial systematic search strategy concentrated on cancer populations, the inclusion criteria were subsequently expanded to encompass a broader range of adult populations and study types relevant to cognitive awareness and anosognosia, including those with Alzheimer’s disease, frontotemporal dementia, traumatic brain injury, mild cognitive impairment, and stroke. This broader approach allowed for the extrapolation of relevant concepts related to anosognosia and self-awareness from a wider neurological context. Studies from the author’s existing bibliography that met these broader inclusion criteria were also incorporated; these were screened and selected using the same pre-defined criteria as the articles found in the database search, ensuring a comprehensive and systematic mapping of the relevant literature.

### Inclusion and exclusion criteria

Publication Type: Peer-reviewed articles, encompassing:

- Original research studies (quantitative, qualitative, or mixed-methods).
- Systematic reviews and meta-analyses.
- Narrative reviews and literature reviews.
- Methodological articles, editorials, commentaries, and recommendation papers that discuss relevant theoretical or practical constructs.

Population: Studies involving adult populations (aged ≥ 18 years) with one of the following conditions:

- Cancer (either non-neurological or neurological cancers, including primary brain tumors and brain metastases).
- Other central nervous system (CNS) conditions where cognitive awareness (e.g., anosognosia, metacognition) is a relevant construct (e.g., Alzheimer’s disease, frontotemporal dementia, traumatic brain injury, mild cognitive impairment, stroke).

Content (Phenomena of Interest): Articles focusing on at least one of the following aspects:

- Assessment of cognitive function using subjective measures (e.g., self-reported complaints, questionnaires).
- Assessment of cognitive function using objective measures (e.g., neuropsychological tests, functional or structural and functional neuroimaging).
- The relationship or discrepancy between subjective and objective cognitive measures.
- The theoretical or clinical construct of self-awareness, metacognition, or anosognosia related to cognitive abilities.
- Neural correlates of cognitive function or awareness, as explored through neuroimaging or other biological markers.

Exclusion Criteria:

- Studies focusing exclusively on pediatric populations (aged < 18 years).
- Publications not available in English.
- Studies focused on pharmacological effects, molecules (e.g., TAU, Amyloid Beta).
- Non-peer-reviewed material such as conference abstracts without full paper publication, unpublished theses, book chapters not subjected to peer review, or articles from non-academic media.
- Studies where the primary focus is not cognitive function or its correlates (e.g., studies dealing exclusively with fatigue, depression, or other symptoms without a cognitive assessment component).

### Study selections process

**Figure 1:**
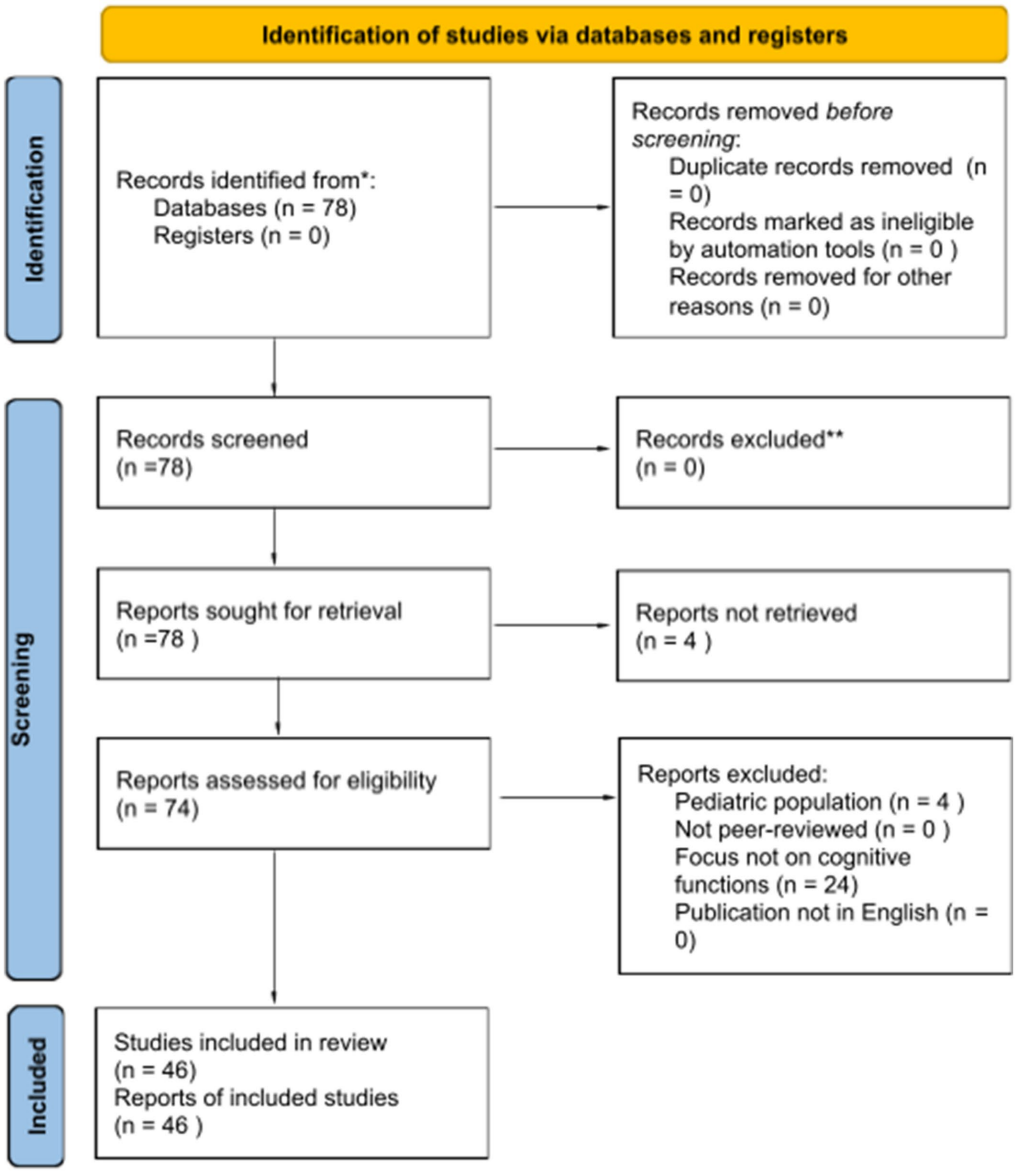
PRISMA flow diagram for the screening and selection process of studies included in this scoping review. *Consider, if feasible to do so, reporting the number of records identified from each database or register searched (rather than the total number across all databases/registers). **If automation tools were used, indicate how many records were excluded by a human and how many were excluded by automation tools.

After duplicate removal, titles and abstracts were screened for relevance by the author. Full-text articles were then reviewed to determine final eligibility based on the predefined inclusion and exclusion criteria. Any uncertainties regarding inclusion were resolved through careful re-evaluation. The selection process is summarized in the PRISMA flow diagram (Figure 1). The final list of 46 studies included in this review is detailed in Table 1.

**Table 1:** Study characteristics of articles included in the scoping review.

First author and year	Study aim/purpose	Study design	Population/participants	Main findings/conclusion
Alvarez J. (2013) (78)	To evaluate the effect of EEG biofeedback as an intervention to reduce post-cancer cognitive impairment.	Clinical Trial/Pilot Study	Cancer survivors with post-cancer cognitive impairment	EEG biofeedback may have a positive effect on reducing cognitive impairment.
Apple A. C. (2017) (92)	To investigate whether subtle hippocampal deformities are associated with reduced episodic memory and self-reported cognitive concerns in breast cancer survivors.	Neuroimaging Study	Breast cancer survivors	Subtle hippocampal deformities are related to poorer episodic memory and subjective cognitive concerns.
Apple A. C. (2018) (55)	To determine if hippocampal functional connectivity is related to self-reported cognitive concerns in breast cancer patients.	Neuroimaging Study	Breast cancer patients undergoing adjuvant therapy	A relationship exists between hippocampal functional connectivity and self-reported cognitive concerns.
Bail J. R. (2020) (74)	To explore the relationship between cancer-related symptoms and adherence to a cognitive intervention.	Mixed-Methods Study	Breast cancer survivors	Cancer-related symptoms, such as fatigue and pain, can negatively impact adherence to cognitive interventions.
Baudino B. (2012) (59)	To investigate the long-term effects of chemotherapy on cognitive functions and brain metabolism.	Neuroimaging Study	Lymphoma patients	Long-term cognitive deficits and alterations in brain metabolism were found in lymphoma patients post-chemotherapy.
Bellens A. (2020) (73)	To evaluate a video-game based cognitive training for breast cancer survivors with cognitive impairment.	Prospective Randomized Pilot Trial	Breast cancer survivors with cognitive impairment	The video game intervention was feasible and showed promising results for cognitive improvement.
Ponto L. L. (2015) (64)	To determine if frontal hypometabolism can be identified using PET scans in elderly breast cancer survivors.	Pilot Study (PET)	Elderly breast cancer survivors	Elderly breast cancer survivors show signs of frontal hypometabolism, suggesting a potential biomarker.
Bruno J. (2012) (54)	To examine resting-state functional brain network topology in chemotherapy-treated breast cancer survivors.	Neuroimaging Study (fMRI)	Chemotherapy-treated breast cancer survivors	Altered brain network topology was observed in the survivors compared to controls.
Carroll J. E. (2019) (15)	To investigate the relationship between cognitive performance and biological aging markers in breast cancer survivors.	Correlational Study	Breast cancer survivors	Cognitive performance is significantly associated with biological markers of aging in this population.
Coro D. G. (2021) (75)	To conduct a feasibility study on the relationship between diet and cognition.	Feasibility Study	Breast and colorectal cancer survivors	Provides preliminary evidence for a link between diet and cognition, supporting the feasibility of a larger study.
de Ruiter M. B. (2011) (56)	To examine cerebral hyporesponsiveness and cognitive impairment long after chemotherapy for breast cancer.	Neuroimaging Study	Breast cancer survivors (10 years post-chemotherapy)	Cognitive impairment and cerebral hyporesponsiveness persist long-term in this population.
Deng G. (2022) (76)	To compare the effects of vigorous vs. restorative yoga on objective cognition functions.	Randomized Controlled Pilot Trial	Sedentary breast and ovarian cancer survivors	The study provides preliminary evidence on the differential impact of yoga types on objective cognition.
Ferguson R. J. (2007) (8)	To investigate brain structure and function differences in monozygotic twins to isolate the effects of breast cancer chemotherapy.	Twin Study	Monozygotic twins with one having undergone chemotherapy	Differences in brain structure and function were observed, suggesting a direct effect of chemotherapy.
Ferguson R. J. (2012) (83)	To develop and test the effectiveness of a CBT-based intervention for chemotherapy-related cognitive change.	Waitlist Control Trial (RCT)	Cancer survivors with chemotherapy-related cognitive change	The CBT-based intervention showed promising results in reducing cognitive complaints.
Gokal K. (2015) (80)	To outline the protocol for a randomized controlled trial to investigate whether physical activity can maintain cognitive functioning and well-being.	Study Protocol (RCT)	Breast cancer patients treated with chemotherapy	Not applicable (protocol).
Gutenkunst S. L. (2021) (61)	To identify correlates of cognitive impairment in adult cancer survivors who have received chemotherapy and report cognitive problems.	Correlational Study	Adult cancer survivors with self-reported cognitive problems	Identifies various demographic, clinical, and psychological factors associated with cognitive impairment.
Hartman S. J. (2018) (84)	To evaluate the effect of a physical activity intervention on both objective and self-reported cognitive functioning.	Randomized Controlled Trial	Breast cancer survivors	The physical activity intervention had a positive effect on both objective and self-reported cognitive functioning.
Hutchinson A. D. (2012) (35)	To systematically review the literature on objective and subjective cognitive impairment following chemotherapy for cancer.	Systematic Review	Cancer patients receiving chemotherapy	There is a general dissociation between objective and subjective measures of cognitive impairment.
Joly F. (2011) (79)	To review the consequences of cognitive dysfunction in terms of cancer disease management.	Narrative Review	N/A	Cognitive dysfunction has a significant impact on treatment adherence and patient autonomy.
Jung M. S. (2017) (24)	To investigate post-treatment cognitive dysfunction in women treated with thyroidectomy for papillary thyroid carcinoma.	Observational/Descriptive Study	Women treated with thyroidectomy for papillary thyroid carcinoma	The study describes the prevalence and characteristics of cognitive dysfunction in this specific population.
Kim R. (2025) (11)	To assess self-reported cognitive function in older breast cancer survivors after chemotherapy treatment.	Cross-sectional/Longitudinal Study	Older breast cancer survivors after chemotherapy	Provides insights into the nature of subjective cognitive complaints in this specific age group.
Klaver K. M. (2024) (88)	To explore the association between neuropsychological test performance, self-reported cognitive function, and work-related outcomes.	Correlational Study	Occupationally active cancer survivors with cognitive complaints	Both objective and subjective cognitive measures are associated with work-related outcomes.
Kobayashi L. C. (2020) (47)	To examine the relationship between pre-treatment cognitive function and subsequent well-being.	Longitudinal Study	Older breast cancer survivors	Pre-treatment cognitive function may serve as a predictor of long-term well-being.
Lee P. E. (2016) (98)	To review evidence on cognitive impairment associated with endocrine treatment in breast cancer survivors.	Literature Review	N/A	Synthesizes the existing evidence, concluding that endocrine therapy can be associated with cognitive impairment.
Mariani M. (2018) (19)	To compare the neuropsychological profiles of breast cancer and brain tumor cohorts.	Comparative Study	Breast cancer and brain tumor cohorts	Reports on distinct neuropsychological profiles in the two groups, highlighting differences in cognitive deficits.
Meneses K. (2018) (93)	To evaluate a speed of processing training program in middle-aged and older breast cancer survivors.	Randomized Controlled Pilot Study	Middle-aged and older breast cancer survivors	The training program showed potential for improving speed of processing in the study population.
Myers J. S. (2019) (81)	To use pupillary response as a measure of cognitive effort in breast cancer survivors.	Experimental/Observational Study	Breast cancer survivors	Breast cancer survivors exhibit increased pupillary response, suggesting greater cognitive effort to complete tasks.
Myers J. S. (2020) (77)	To conduct a pilot study of a telehealth-delivered cognitive rehabilitation intervention.	Controlled Pilot Study	Breast cancer survivors	The telehealth intervention for cognitive rehabilitation was feasible and showed promising preliminary results.
Nakamura Z. M. (2024) (82)	To systematically review the impact of cognitive rehabilitation on cognitive and functional outcomes.	Systematic Review	Adult cancer survivors	Cognitive rehabilitation interventions can be effective in improving cognitive and functional outcomes.
O’Farrell E. (2013) (38)	To review the current understanding of “chemo fog.”	Narrative Review	N/A	Summarizes the state of the research on chemotherapy-related cognitive impairment (CRCI) at the time.
O’Farrell E. (2016) (99)	To examine whether using change measures can help reconcile the objective-subjective disparity in cognitive impairment.	Correlational Study	Cancer survivors with cognitive impairment	The study investigates the dissociation between objective and subjective measures, suggesting that change scores might provide a more nuanced understanding.
Paquet L. (2018) (18)	To test a novel account of the dissociation between self-reported memory problems and objective memory performance.	Correlational Study	Chemotherapy-treated breast cancer survivors	The study provides a theoretical framework to explain the objective-subjective discrepancy in cognitive function.
Parada H. (2023) (48)	To assess neurocognitive test performance after cancer in a specific Hispanic/Latino cohort.	Cohort Study	Middle-aged and older Hispanic/Latino adults with cancer	The study provides valuable data on cognitive outcomes in a historically underrepresented population.
Pinheiro V. H. G. (2025) (85)	To conduct a systematic review and meta-analysis on the effect of exercise on cognitive function.	Systematic Review & Meta-analysis	Breast cancer survivors	Exercise has a positive effect on cognitive function in breast cancer survivors.
Player L. (2014) (1)	To explore women’s experiences of ‘chemobrain’ and the potential role of occupational therapy.	Qualitative Study	Women treated for breast cancer	Provides insights into the lived experience of cognitive changes and suggests a role for occupational therapy.
Smith T. M. (2021) (9)	To compare a standard computer-assisted cognitive training program with a music-enhanced version.	Mixed-Methods Study	Cancer survivors with cognitive impairment	The study reports on the comparative effectiveness and participant experiences of the two training programs.
Tan C. J. (2020) (14)	To describe self-reported cognitive outcomes in adolescent and young adult patients with non-CNS cancers.	Descriptive Study	Adolescent and young adult cancer patients	The study provides an overview of self-reported cognitive challenges in this understudied population.
Van Dyk K. (2022) (94)	To use machine learning to associate persistent self-reported cognitive decline with neurocognitive decline.	Longitudinal Study (with Machine Learning)	Older breast cancer survivors	Machine learning was used to identify associations and predict objective decline from subjective reports.
Vardy J. (2007) (10)	To review the literature on cognitive function after chemotherapy in adults with solid tumors.	Literature Review	Adults with solid tumors	Provides a comprehensive overview of the research on cognitive function in this population at the time.
Veal B. M. (2023) (67)	To examine the associations between subjective cognition, daily memory lapses, objective performance, fatigue, and depressed mood.	Correlational Study	Breast cancer survivors	The study highlights the complex relationships between subjective cognitive complaints, daily life struggles, and mood.
Vega J. N. (2018) (100)	To compare self-reported chemotherapy-related cognitive impairment with cognitive complaints following menopause.	Comparative Study	Cancer survivors with CRCI vs. women with menopausal cognitive complaints	The study found similarities in self-reported cognitive complaints between the two groups.
Von Ah D. (2015) (36)	To evaluate relationships between self-reported cognitive function, objective cognitive performance, and other symptoms.	Correlational Study	Breast cancer survivors	Reports on the correlations between self-reported cognitive function and other clinical measures.
Von Ah D. (2018) (95)	To investigate the relationship between self-reported cognitive function and work-related outcomes.	Correlational Study	Breast cancer survivors	Self-reported cognitive function is significantly associated with work-related outcomes like absenteeism and productivity.
Wang L. (2016) (17)	To examine prefrontal activation during memory tasks and its relation to patient-reported cognition.	Neuroimaging Study (fMRI)	Cancer survivors post-chemotherapy	Found reduced prefrontal activation during memory tasks, which was correlated with impaired patient-reported cognition.
Yamamoto S. (2020) (26)	To describe self-reported cognitive decline in Japanese patients treated with endocrine therapy.	Descriptive Study	Japanese breast cancer patients on endocrine therapy	Provides data on the prevalence and characteristics of cognitive complaints related to endocrine therapy in a specific cultural context.
Zeng Y. (2017) (39)	To examine the relationship between subjective cognitive impairment and brain structural networks.	Cross-sectional Study	Chinese gynaecological cancer survivors	Found an association between subjective cognitive impairment and alterations in brain structural networks.

### Data extraction and synthesis

Data were extracted on study design, cancer population, treatment type, cognitive domains assessed, methods for measuring subjective and objective cognition, tools for evaluating self-awareness, and main findings. The results were synthesized narratively, with special attention to patterns of discrepancy, theoretical interpretations, and clinical relevance.

### Synthesis of results

#### The discrepancy between subjective and objective cognitive performance

A central theme across the literature is the recurrent discrepancy between self-reported cognitive difficulties and objective neuropsychological performance in cancer survivors. Numerous studies confirm that many patients show complain of memory, attention, or executive dysfunction despite performing within normal limits on standardized tests (2, 5, 6, 22, 35–39). Earlier work by Lange et al. (13) highlighted the frequent discrepancies of subjective complaints from neuropsychological test results, suggesting that such mismatches may emerge even in the absence of measurable deficits. In this study it has been shown that cancer-related cognitive complaints started especially during chemotherapy for 35% of the sample; 30% after chemotherapy; 15% during hormone therapy and lasted about 2 years, with an important impact on work resumption. Conversely, others show measurable cognitive impairments but fail to acknowledge or report them (7, 8, 13, 38, 40, 41). This bi-directional mismatch suggests the presence of impaired self-awareness mechanisms similar to anosognosia, as described in neurological contexts such as early-stage Alzheimer’s disease, where similar patterns of impaired insight have been observed (42–44). Despite the frequency of subjective-objective mismatch, only a limited number of studies operationalize or directly measure self-awareness (35, 45, 97) A minority compare subjective ratings with objective tests to derive discrepancy scores (3, 6, 46). Others use interviews or qualitative methods (7, 35), while very few attempt longitudinal or imaging-based approaches to assess awareness over time (47, 48). Longitudinal studies, such as Menning et al. (49), further confirm that discrepancies between subjective and objective cognition may persist or evolve over time, independent of treatment status. Other discrepancies between subjective-objective cognitive performance have been shown by Von Ah et al. (27) which reported that subjective cognitive concerns often diverge from neuropsychological results, echoing findings across various cancer populations. In support of this pattern, Tan et al. (14) found that young non-Central Nervous System (CNS) tumor survivors reported significantly higher levels of self-reported cognitive difficulties than healthy controls, while maintaining neuropsychological performance within normal limits on standardized tests. This two-way mismatch between perceived and measured cognitive function mirrors characteristics of anosognosia, extensively studied in neurological and neuropsychological conditions, yet remains a relatively novel concept in psycho-oncology. Recent studies have begun to explore this discrepancy in cancer survivors, highlighting its clinical relevance and the need for integrative models that account for both subjective experience and objective impairment (36, 39, 50, 51). This consistent finding highlights that the subjective-objective discrepancy is a core feature of CRCI and is not merely an artifact of measurement, but rather a complex phenomenon with underlying mechanisms that warrant further investigation.

#### Neurobiological features

To understand the mechanisms behind the subjective-objective cognitive discrepancy, it is crucial to explore the underlying neurobiological and psychological factors. Despite methodological limitations, a number of hypotheses have emerged regarding the mechanisms contributing to impaired insight. From a neuroimaging point of view, structural and functional brain alterations, particularly in default mode network and midline cortical structures involved in self-appraisal, have been proposed (37, 41, 52, 53) (see Supplementary material). Moreover, Bruno et al. (54) reported both structural and functional alterations. More precisely, they highlighted reductions in grey and white matter in brain regions associated with cognitive functions, including the frontal and temporal lobes, as well as subcortical structures, in cancer survivors compared to healthy controls. Additionally, they reported disrupted functional connectivity among the dorsolateral prefrontal cortex (DLPFC), orbitofrontal cortex (OFC), hippocampus, and parahippocampal gyrus. Interestingly, breast cancer survivors reported increased cognitive complaints, as measured by subjective self-report questionnaires such as the Behavioral Rating Inventory of Executive Function (BRIEF) and the Multifactorial Memory Questionnaire (MMQ). However, these self-reported scores did not correlate with performance on objective neuropsychological tests, indicating a disconnection between perceived and objectively measured cognitive functioning. Apple et al. (55) identified functional alterations in hippocampal connectivity, particularly with the precuneus and cortical midline structures, that were selectively associated with self-reported cognitive concerns but not with objective performance, offering neuroimaging-based support for a dissociation between subjective and objective cognition in breast cancer survivors (55, 56). Likewise, Henneghan and Kesler (50) have identified salience network connectivity reductions correlating with subjective complaints. These findings, along with neurodegenerative models like Hanseeuw et al. (57), offer direct paths for integrating neurobiological models into CRCI awareness research (50, 55, 57, 58). Similarly, Wang et al. (17) found that oncology patients treated with adjuvant chemotherapy showed reduced activation in the right DLPFC during working memory tasks and in the left-middle hippocampus during visual recognition tasks. These patterns suggest under-recruitment of brain regions involved in memory and cognitive control. The study by Baudino et al. (59) aligns with these features. They showed that individuals who underwent chemotherapy exhibited hypometabolism in prefrontal areas, as well as in the cerebellum, medial cortices and limbic brain regions. This hypometabolism was associated with poorer performance on neuropsychological tests, including the Mini-Mental State Examination (MMSE), Trail Making Test-B (TMT-B), and verbal fluency tasks. Despite all these neuroimaging findings, there was no direct correlation with patient-reported cognitive complaints. Nonetheless, patients reported more cognitive difficulties and reduced quality of life. Behavioral performance was mostly comparable to controls, though slight declines emerged with increased task difficulty. The authors interpret the reduced DLPFC and hippocampal activity as evidence of chemotherapy-related neural dysfunction, consistent with prior findings of structural brain changes and white matter damage. Neuroimaging research by Kesler et al. (60) revealed altered local brain network connectivity in breast cancer survivors, precisely in temporal, frontal-temporal and temporal–parietal areas, highlighting distinct associations between subjective cognitive complaints and objective cognitive function, thus providing evidence for neurobiological mechanisms underlying impaired self-awareness. However, evidence suggests that the subjective-objective cognitive discrepancy is not only neurobiological in nature. Psychological factors such as fatigue and depressive symptoms are strongly correlated with self-reported cognitive complaints, suggesting that the subjective experience of cognitive deficit is profoundly influenced by the patient’s psychological well-being (61). In summary, these neuroimaging studies provide a crucial neurobiological basis for the subjective-objective cognitive discrepancy, demonstrating that structural and functional brain alterations, particularly in regions involved in self-appraisal, may directly contribute to impaired self-awareness in cancer survivors.

#### Clinical implications and non-pharmacological interventions

The interplay of neurobiological and psychological factors has significant clinical implications for patient care and the planning of interventions. However, the interaction between these correlates of awareness remains largely unexplored in cancer populations (62, 63). Neuroinflammation, disrupted connectivity and damage to frontoparietal circuits may impair metacognitive monitoring (2, 22, 64, 65). Psychological factors such as anxiety, avoidance, or cognitive reappraisal strategies may also lead to over or under-reporting (13, 16, 41). For example, Hansen et al. (66) emphasized how self-reported memory problems may poorly correlate with objective measures, underscoring the need to consider psychological and contextual factors in assessment (66, 67). Similarly, Gutenkunst et al. (61) reported that psychological factors, such as mood, anxiety, stress, were correlated with self-reported cognitive impairment. The clinical significance of self-reported cognitive symptoms is confirmed by Maeir et al. (68), who identified the severity of cognitive complaints, fatigue, and depressive symptoms as predictors of worse quality of life in a sample of adults with CRCI. This suggests that, beyond objective findings, subjective perceptions have a real impact on patients’ daily lives. Awareness of cognitive deficits plays a key role in treatment adherence, occupational functioning, and psychosocial adaptation. Patients unaware of their impairments may underestimate the need for cognitive rehabilitation or support (35, 69), while over-reporting may reflect distress or comorbid depression rather than true decline (6, 7, 70). Several authors highlight the importance of integrating awareness measures into survivorship care planning (3, 6, 46, 53, 71, 72). Furthermore, for the acknowledged cognitive impairment in this population, many studies have been conducted focusing on the effects on the cognition due to no-pharmacological treatments: such as food, video games, music, home based training, cognitive behavioral therapy (CBT), psychoeducation-based cognitive rehabilitation intervention, physical exercise, yoga, electroencephalogram Biofeedback (9, 73–85). These studies provided valuable alternatives to take into account for improving cognitive functioning in individuals with CRCI. In conclusion, the awareness of cognitive deficits plays a critical role in patient outcomes, influencing adherence to interventions and overall quality of life. The development of both pharmacological and non-pharmacological treatments, and their integration with awareness-based strategies, represents a key area for future research and clinical practice.

#### Methodological challenges and conceptual gaps

Despite the significant clinical and neurobiological evidence discussed, the field of cognitive self-awareness in cancer survivors faces substantial methodological and conceptual challenges. Very few studies assess awareness with validated tools, and those that do often fail to contextualize the findings within established neurological models of anosognosia (47, 48, 69). We can affirm how the most consistent finding across studies was a frequent mismatch between subjective complaints and objective cognitive performance (3, 35, 53, 70). Importantly, these mismatches are not merely noise: they reflect deeper issues of insight, self-monitoring, and the brain’s capacity to represent its own cognitive state (37, 52). Yet, despite its clinical relevance, very few studies directly assess metacognitive insight. Most rely on parallel administration of subjective and objective tools, without conceptualizing the gap between them as a marker of impaired awareness. Even fewer adopt structured methods, validated discrepancy indices, or neuroimaging-informed frameworks. This methodological inconsistency makes it difficult to compare findings or assess prevalence across samples (13, 41, 53). A review article by Wefel et al. (58) highlighted the frequent occurrence of cognitive complaints in cancer survivors despite the absence of objective deficits in many cases, underscoring the methodological challenges in assessing cancer-related cognitive impairment and the need for more standardized and multidimensional evaluation approaches (58). The conceptual vacuum is matched by a theoretical one: CRCI is rarely discussed within models of self-appraisal or cognitive insight developed in other clinical populations (69). While neurological studies underscore the involvement of cortical midline structures, default mode network dysfunction, and fronto-parietal dysconnectivity in anosognosia, few CRCI investigations apply these frameworks to cancer populations. Clinically, unawareness of cognitive decline may significantly influence care. Patients who are unaware of their deficits may underutilize supportive interventions, mismanage medication, or fail to recognize cognitive risk in return-to-work decisions (7, 70, 79). Conversely, exaggerated cognitive complaints may reflect affective distress or indicate a need for psychological, rather than cognitive, intervention (7, 70). Supporting this, Mehnert et al. (86) found that although neuropsychological tests did not consistently reveal impairments, nearly half of the patients reported subjective cognitive deficits, which were strongly associated with high levels of fatigue and decreased quality of life. This has direct implications for triage, resource allocation, and survivorship care planning. To conclude, our findings suggest an urgent need to develop and validate CRCI-specific tools for awareness assessment, ideally grounded in neurological and psychiatric models of self-monitoring. Moreover, integrating awareness metrics into longitudinal designs could help clarify their evolution over time and their responsiveness to intervention.

## Discussion

This scoping review systematically mapped current knowledge regarding the self-awareness of cognitive deficits in cancer survivors, highlighting an emerging yet fragmented field. While cancer-related cognitive impairment (CRCI) has received growing attention, its metacognitive dimensions, specifically, the awareness or unawareness of such impairment, remain understudied and inconsistently addressed. The most consistent finding across studies was a frequent mismatch between subjective complaints and objective cognitive performance (3, 5, 18, 35, 87). This bidirectional discrepancy resembles patterns observed in anosognosia, a phenomenon widely documented in neurology and neuropsychology but only recently considered in psycho-oncology (69). Importantly, these mismatches are not merely noise: they reflect deeper issues of insight, self-monitoring, and the brain’s capacity to represent its own cognitive state. Yet, despite its clinical relevance, very few studies directly assess metacognitive insight. Most rely on parallel administration of subjective and objective tools, without conceptualizing the gap between them as a marker of impaired awareness. Even fewer adopt structured methods, validated discrepancy indices, or neuroimaging-informed frameworks. This lack of operational consistency makes it difficult to compare findings or assess prevalence across samples (13, 41, 87). CRCI is rarely discussed within models of self-appraisal or cognitive insight developed in other clinical populations. While neurological studies emphasize the role of cortical midline structures, default mode network disruption, and fronto-parietal disconnection in anosognosia (40, 52) few CRCI studies have leveraged these models to explain awareness deficits. This represents a missed opportunity for translational integration. Clinically, unawareness of cognitive decline may significantly influence care. Patients who are unaware of their deficits may have more difficulties in the use of cognitive strategies, supportive interventions and to solve work activities (5, 7, 88). Conversely, exaggerated complaints may mask affective distress or signal a need for psychological (and not cognitive) intervention (6). This has direct implications for triage, resource allocation, and survivorship care planning. Our findings suggest an urgent need to develop and validate CRCI-specific tools for awareness assessment, ideally grounded in neurological and psychiatric models of self-monitoring. Moreover, integrating awareness metrics into longitudinal designs could help clarify their evolution over time and their responsiveness to intervention.

### Underlying pathophysiological factors influencing objective vs. subjective CRCI and self-awareness

As fully described in the article, it is clear that CRCI is multifactorial. The several factors that constitute CRCI may influence the objective performance, subjective perception and anosognosia. Briefly, in this section the authors want underscore the factors that can influence this construct are:

1. Paraneoplastic syndromes: the immune-mediated neurotoxic effects associated with tumor presence, may disrupt cortical and subcortical circuits involved in cognition and metacognitive monitoring, potentially resulting in impaired insight or anosognosia (89).
2. Pre-treatment cognitive impairment: cognitive dysfunction may be present (already) before any oncological therapy due to tumor, effects of neoplasia, or comorbid conditions (e.g., vascular or metabolic disease), influencing both cognitive performance and awareness accuracy (11).
3. Off-target and neurotoxic effects of oncological and supportive treatments: chemotherapy, immunotherapy, endocrine therapy, corticosteroids, and analgesics may differentially affect brain networks responsible for executive control and self-monitoring, leading to dissociations between performance and self-perception (17)
4. Immune responses and neuroinflammatory mechanisms: dysregulated immune activation—including tumor-associated inflammation, cytokine release, microbiome alterations, and immune-related neurotoxicity post allo-HSCT—may alter neurobiological substrates linked to metacognition and interoceptive accuracy (90, 91).
5. Tumor-associated systemic inflammation: chronic low-grade inflammation can impair neural circuits such as the default mode network (DMN), salience network (SN), and fronto-parietal networks (FPN), which are crucial for both cognitive performance and internal awareness of deficits (50).
6. Perceptual discrepancies in CRCI: patients may prioritize lived cognitive difficulties emerging in daily functioning (e.g., overload, multitasking failures) rather than standardized test performance, contributing to subjective-objective mismatch (13).
7. Individual cognitive appraisal and psychological processing: anxiety, depression, hypervigilance, denial, coping style, and metacognitive beliefs can modulate perceived impairment, leading to amplification (hyper-awareness) or minimization (anosognosic tendencies) of cognitive issues (68).
8. Disease-, therapy-, and patient-related modifiers: cancer type, treatment intensity, cognitive reserve, age, fatigue, sleep quality, hormonal status, and resilience may alter both the emergence of CRCI and the individual’s capacity to detect and interpret changes in cognitive function (47).
9. Monitoring and psycho-oncological interventions: the extent to which cognitive performance is objectified (e.g., frequency of testing, feedback mechanisms) and integrated into psycho-oncological support may influence awareness, especially in longitudinal care pathways (e.g., HL or breast cancer populations assessed pre- and post-treatment).
10. Biopsychosocial interdependence and network-based multicausality: the interplay among biological, psychological, and social factors suggests that CRCI-related anosognosia does not emerge from a single etiological source but from networked dysfunctions that influence insight in a nonlinear manner (2).
11. Longitudinal trajectories and evolving self-awareness: awareness of cognitive changes may fluctuate over time as patients adapt, reattribute, or normalize cognitive alterations, highlighting the need for longitudinal evaluation of anosognosia in CRCI (57).

To conclude, these factors suggest how CRCI arises from a link between neurobiological and psychological processes that dynamically can influence both cognition and self-awareness. Understanding the interactions is essential for developing tailored interventions, recognizing and addressing cognitive performance and metacognitive accuracy.

### Future directions

The findings of this review underscore an urgent need to conceptually and methodologically elevate the study of cognitive self-awareness in cancer survivors. While the discrepancy between subjective complaints and objective impairment has been acknowledged across multiple studies, it has rarely been explicitly framed as a metacognitive phenomenon or studied within theoretical models of anosognosia. First (i), future research should aim to operationalize awareness of CRCI more systematically. Standardized discrepancy indices, insight questionnaires, and performance-prediction paradigms, as used in other clinical populations, could provide more precise and clinical measures of self-awareness. Validating such tools in oncology populations would allow for clearer diagnostic thresholds and longitudinal tracking. Second (ii), neurobiological investigations of self-awareness in CRCI remain sparse. Studies that use structural and functional imaging, particularly those examining cortical midline structures, default mode network connectivity, and fronto-parietal circuits, could clarify the neural mechanisms underlying impaired. Third (iii), greater attention should be paid to the functional consequences of impaired awareness. Longitudinal and interventional studies are needed to examine whether poor insight affects treatment adherence, occupational functioning, or psychosocial adjustment. It is also essential to differentiate patients who underreport deficits due to lack of awareness from those who overreport due to anxiety, hypervigilance, or depressive symptomatology (5, 7, 45). Fourth (iv), we recommend that psychosocial and rehabilitative interventions explicitly assess and target both self-awareness and cognitive functions. Interventions that enhance metacognitive monitoring, such as self-reflection training, feedback-based strategies, and compensatory cognitive rehabilitation, may be especially beneficial for survivors with impaired awareness. Their efficacy, however, remains to be systematically tested in this population. Finally, a stronger integration between oncology and neuropsychology is required. CRCI-related anosognosia is a cross-disciplinary phenomenon, requiring collaboration between oncologists, neuropsychologists, neuroscientists, and rehabilitation specialists. Developing a shared language, common metrics, and integrated models will be key to advancing this field.

### Limitations

To the best of our knowledge, despite offering the first systematic scoping review on self-awareness of cognitive impairment in cancer survivors, this study presents several limitations that warrant discussion. First, although our literature search was conducted using a comprehensive strategy on PubMed and limited to the years 2000–2025, the final inclusion of only 46 articles may reflect a combination of strict eligibility criteria and limitations in keyword selection. The narrow focus on studies that explicitly examined subjective-objective cognitive discrepancy or metacognitive constructs may have led to the exclusion of relevant but tangential studies. Additionally, the lack of standardized terminology in this area (e.g., “insight,” “awareness,” “subjective complaints”) may have hindered retrieval of pertinent articles.

Second, we did not register a review protocol *a priori* (e.g., with the Open Science Framework or PROSPERO), which reduces transparency in study design and may introduce bias in study selection or synthesis. Third, the scoping review design itself, while appropriate for mapping a fragmented field, does not allow for formal quality assessment or meta-analytic conclusions. The heterogeneity in populations, methodologies, and awareness measures across studies further limits the ability to generalize findings or draw strong inferential claims.

Finally, language and database restrictions, limiting our search to English-language articles and using only PubMed, may have omitted studies indexed elsewhere or published in other languages. To strengthen future reviews, we recommend refining and expanding the keyword strategy and using multiple databases (e.g., PsycINFO, Scopus, Embase). Search terms such as “cognitive insight,” “awareness of deficits,” “anosognosia,” “subjective cognitive decline,” “metacognition,” and “self-awareness AND cancer” could yield a broader evidence base.

## Conclusion

This scoping review represents an initial step toward synthesizing knowledge on self-awareness of cognitive impairment in cancer survivors: a clinically and theoretically relevant but underexplored domain. Our findings suggest that while the discrepancy between subjective and objective cognitive functioning is well-documented in oncology, few studies conceptualize this as a metacognitive or anosognosic phenomenon. Furthermore, the field lacks standardized tools to measure awareness, limiting both clinical assessment and research comparability. Impaired cognitive self-awareness in cancer survivors may influence treatment adherence, quality of life, and response to rehabilitation. Understanding and addressing this phenomenon has the potential to improve survivorship care and personalized intervention planning. However, current evidence is fragmented, methodologically inconsistent, and heavily reliant on indirect proxies. To move the field forward, future studies must adopt more rigorous and theoretically grounded approaches. This includes validating instruments for assessing awareness, integrating neuroimaging to explore its neural correlates, and designing interventions that target metacognitive deficits. More interdisciplinary collaboration will be critical to building a coherent research agenda. In sum, CRCI-related anosognosia represents a clinically meaningful construct deserving greater empirical and theoretical attention. By laying the groundwork for future investigations, this review highlights the need to move beyond detection of cognitive complaints toward a more nuanced understanding of how survivors perceive and misperceive their own cognitive functioning.

## Data Availability

The original contributions presented in the study are included in the article/Supplementary material, further inquiries can be directed to the corresponding author.
