# Supplementary figures and images for: Mapping self-awareness of cancer-related cognitive impairment: a scoping review of evidence, methods, and neurobiological correlates
Source: Front Neurol. 2025 Dec 11;16:1662935. doi: 10.3389/fneur.2025.1662935 (PMC12738308; doi:10.3389/fneur.2025.1662935)

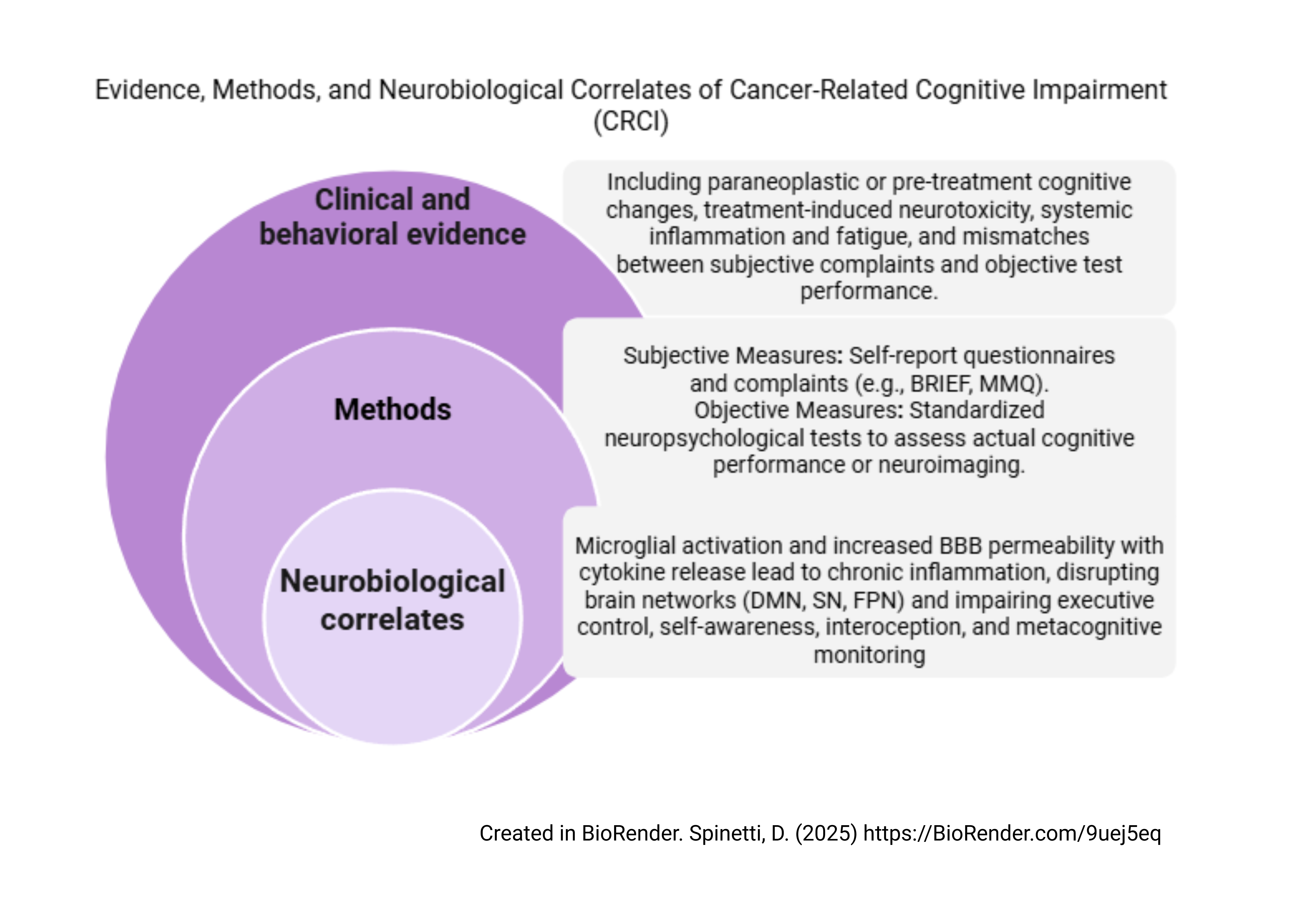

Supplement: Supplementary file 1 [file Image_1.png]
